# Reduced Incidence of Stroke in Patients with Gout Using Benzbromarone

**DOI:** 10.3390/jpm12010028

**Published:** 2022-01-02

**Authors:** Sheng-Wen Niu, Chi-Chih Hung, Hugo Y. -H. Lin, I-Ching Kuo, Jiun-Chi Huang, Jiun-Shiuan He, Zhi-Hong Wen, Peir-In Liang, Yi-Wen Chiu, Jer-Ming Chang, Shang-Jyh Hwang

**Affiliations:** 1 Graduate Institute of Clinical Medicine, College of Medicine, Kaohsiung Medical University, No. 100, Tzyou 1st Road, Kaohsiung 807, Taiwan; 950138kmuh@gmail.com; 2 Department of Internal Medicine, Kaohsiung Municipal Ta-Tung Hospital, Kaohsiung 807, Taiwan; yukenlin@yahoo.com.tw (H.Y.-H.L.); 980135kmuh@gmail.com (I.-C.K.); kmtth8079@gmail.com (J.-S.H.); 3 Department of Internal Medicine, Division of Nephrology, Kaohsiung Medical University Hospital, Kaohsiung Medical University, Kaohsiung 807, Taiwan; chichi@kmu.edu.tw (C.-C.H.); karajan77@gmail.com (J.-C.H.); chiuyiwen@gmail.com (Y.-W.C.); jemich@kmu.edu.tw (J.-M.C.); 4 Department of Medicine, College of Medicine, Kaohsiung Medical University, Kaohsiung 807, Taiwan; 5 Department of Marine Biotechnology and Resources, National Sun Yat-Sen University, Kaohsiung 807, Taiwan; wzh@mail.nsysu.edu.tw; 6 Department of Pathology, Kaohsiung Medical University Hospital, Kaohsiung Medical University, Kaohsiung 807, Taiwan; peirinl@yahoo.com

**Keywords:** benzbromarone, stroke, gout

## Abstract

Gout is strongly associated with the incidence of atherosclerotic events, including stroke and myocardial infarction. Considering the increased prevalence of stroke in the population with gout, the aim of this study was to evaluate the effects of benzbromarone, a uricosuric agent, on the incidence of stroke in the population with gout. We used data from the Taiwanese National Health Insurance Registration Database (NHIRD). The benzbromarone user cohort included 15,143 patients; each patient was age- and sex-matched with one non-user randomly selected from the population with gout. Cox proportional hazard regression analysis was conducted to estimate the effects of benzbromarone on the incidence of stroke in the population with gout. The incidence of stroke was significantly lower in benzbromarone users than in benzbromarone non-users. The HR for the incidence of stroke was lower in male benzbromarone users than in non-users. An analysis of three age groups (<40, 40–59, and ≥60 years) indicated that the HRs in those aged 40–59 years and ≥60 years were significantly lower among benzbromarone users than non-users. In the population with gout, the incidence of stroke was lower in benzbromarone users than in benzbromarone non-users.

## 1. Introduction

Gout is strongly linked to several factors, including the incidence of atherosclerotic events or cardiovascular events (CVE) (including coronary artery disease (CAD) and cerebrovascular events (CVD)), hypertension, obesity, type 2 diabetes mellitus (DM), metabolic syndrome (MetS), chronic kidney disease (CKD), lifestyle factors, and the increased use of causative medications [1]. A study of 232,592 patients demonstrated that gout carries a risk equivalent to that associated with DM for incident stroke, and patients having both gout and DM have a greater risk than those with DM alone for both incident stroke and myocardial infarction (MI) [2]. Gout was observed to be associated with an increased risk of stroke, including ischemic, hemorrhagic, and unspecified stroke [3]. One large study that used data from the Third National Health and Nutrition Examination Survey with 15,773 participants revealed that patients with gout had a 40% increase in CVE, including stroke, and a 50% increase in mortality [4]. Another study demonstrated that serum uric acid was related to a 31% increased risk of future ischemic cerebrovascular events in men and with all-cause mortality in both sexes. Gout may be considered a possible risk factor for CVE because patients with both tophaceous and nontophaceous gout exhibit platelet hyperactivity, and acute gout flares exacerbate platelet activation [5].

Initiating urate-lowering therapy (ULT) may reduce the risk of stroke in patients with gout. A study reported that the prolonged use of ULTs in patients with gout for UA level reduction could decrease inflammation and pro-thrombotic mechanisms and reduce the risk of CVEs, including stroke [6]. One meta-analysis suggested that hyperuricemia may modestly increase the risks of both incident stroke and mortality [7]. However, the meta-analysis study of the cardiovascular effects of ULAs revealed no apparent benefits of CVEs with regard to ULAs, and there is a lack of studies about uricosuric agents [8]. Uricosuric agent use can decrease the risk of DM, possibly because the increased clearance of uric acid (CrUA), and CrUA appears to decrease in proportion to the increases of insulin resistance (IR) and serum uric acid (UA) concentrations [9]. In patients with acute stroke and accompanying elevated serum *low-density lipoprotein* cholesterol (LDL-C) and triglyceride (TG) levels, there was a high prevalence of hyperuricemia [10]. To investigate the effect of benzbromarone on the incidence of stroke, we conducted this study by comparing the clinical outcomes between benzbromarone users and non-users by using Taiwanese NHIRD, which is one of the largest nationwide health registration databases, and initiated from a single-payer program of National Health Insurance (NHI) since 1 March 1995.

## 2. Materials and Methods

### 2.1. Data Sources

The data used in the present study were received from the Longitudinal Health Insurance Database 2000 (LHID2000), which is a subset of the NHIRD that includes all claims data (from 1996 to 2010) for one million beneficiaries. This sample was selected randomly and systematically in 2000. There were no significant differences in age, sex, or healthcare costs between the sample group and all enrolled in the NHI program. The LHID2000 uses International Classification of Diseases, Ninth Revision, Clinical Modification (ICD-9-CM) codes for diagnoses and procedures, details of prescriptions, registry in the Catastrophic Illness Patient Database, and costs covered and paid for by the NHI; it also provides encrypted patient identification numbers, sex, date of birth, and dates of admission and discharge. The Institutional Review Board of Kaohsiung Medical University Hospital approved the protocol of this study (KMUHIRB-EXEMPT(I)-20190390). Informed consent was not required because the datasets were devoid of identifiable personal information.

### 2.2. Study Sample

**Figure 1:**
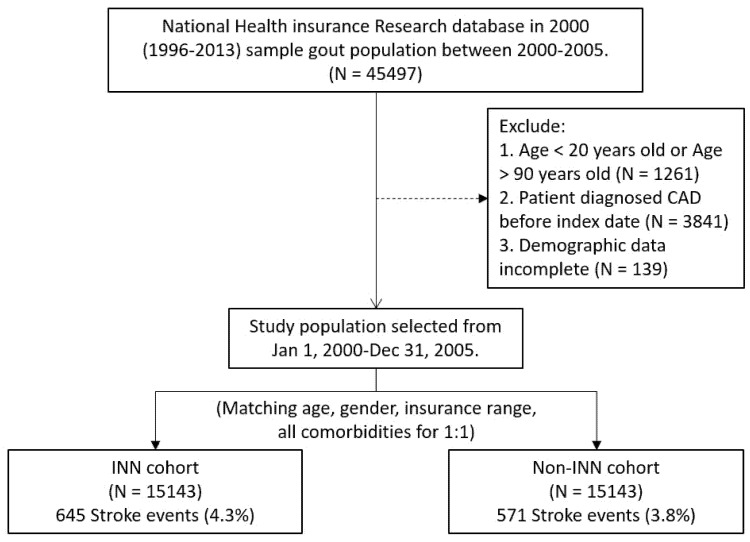
Flow chart of study population.

In order to conduct a retrospective cohort study, a benzbromarone user group and a matched benzbromarone non-user control group were selected during the recruitment period of 2000–2005 (Figure 1). When the patient has: 1. At least two outpatient service claims in any hospital or local medical clinic, the Anatomical Therapeutic Chemistry (ATC) code is AB03 (ICD-9-CM code 274), or 2. The diagnostic code listed in the claim with gout and use of benzbromarone in any hospitalization will be defined as a user of benzbromarone. Patients who were diagnosed with a stroke before 2000, were younger than 20 years, and had incomplete demographic data were excluded. Since the outcome of interest was a new stroke, any patients who were diagnosed with a stroke before the index date (ICD-9-CM code 430–438) were excluded. For each benzbromarone user, a benzbromarone non-user was randomly selected from the data set as a control match. Benzbromarone users and control group members were matched by age, gender, hypertension, DM, dyslipidemia, CKD, CAD, residential area, monthly income, and index date. For each benzbromarone control patient, an index date was created based on the date of their first registration.

Demographic data, including sex, age, geographic area of Taiwan, and monthly income (recorded in NT$), were collected. Baseline comorbidities of these patients were recorded as hypertension (ICD-9-CM codes 401–405), DM (ICD-9-CM codes 250), dyslipidemia (ICD-9-CM code 272), CKD (ICD-9-CM codes 585), and CAD (ICD-9-CM codes 410–414), which were all included because they are known to affect the risk of CVD. The definition of dyslipidemia included 1. LDL-C ≥ 190 mg/dL without any risk factor, 2. total cholesterol (TC) ≥ 240 mg/dL or LDL-C ≥ 160 mg/dL with one risk factor, 3. TG ≥ 200 mg/dL or LDL-C ≥ 130 mg/dL with 2 risk factors, 4. TG ≥ 160 mg/dL or LDL-C ≥ 100 mg/dL with “DM or CVE (including CAD and CVD)”, 5. TG ≥ 500 mg/dL, and 6. TG ≥ 200 mg/dL with “high-*density lipoprotein* cholesterol (HDL-C) < 40 mg/dL or (TC/HDL-C) >5”. The risk factors included: 1. hypertension; 2. male sex; and ≥ 45 years old or female sex and ≥ 55 years old or postmenopausal, 3. a family history of early CVE (male ≤ 55 years old, female ≤ 65 years old), 4. HDL-C < 40 mg/dL; and 5. smoking [11]. We calculated any of these comorbid conditions if the condition was diagnosed in an inpatient setting or represented by three or more ambulatory care claims coded 1 year before the index medical care date. The follow-up duration in person-years (PY) was counted for each person until the diagnosis of CVD, death, or the end of 2005.

### 2.3. Measurements of Benzbromarone

The commercially available benzbromarone (ATC code M04AB03) in Taiwan was analyzed. According to the total supply in days and the quantity of benzbromarone, we counted the cumulative defined daily dose (DDD) of benzbromarone for each benzbromarone user. For benzbromarone, the cumulative DDD, defined by the ATC/DDD system of the WHO Collaborating Center for Drug Statistics and Methodology, was partitioned into three levels at the 33rd and 67th percentiles. Appropriate ATC code and DDD link each product. The assumed average daily maintenance dose of the drug for the main indication for adults is defined as DDD. In order to be able to compare between populations, we use DDD, a fixed unit of measurement that can provide independent price and dosage form and can standardize drug dosages and compare multiple types of drugs. The total amount of drug by the amount of drug in DDD was used to calculate the number of DDDs. The cumulative DDD, representing the dose and duration of exposure, can be used to estimate the sum of the DDDs dispensed by benzbromarone. Therefore, we can correlate the use of benzbromarone with the risk of new-onset strokes in gout patients by using cumulative DDD.

### 2.4. Statistical Analysis

Statistical analysis was performed with SPSS software, version 19 (IBM, Armonk, NY, USA). Comparisons in the study and control cohorts were executed utilizing Pearson’s chi-square test. Subjects of comparison were baseline characteristics, comorbidities, and sociodemographic statuses. The incidence rate in our model is designed as the number of new-onset stroke cases discovered during follow-up divided by the total PY for each group classified into different ages and durations. The ratio of the incidence rate of new-onset stroke between the study and control cohorts was estimated using the Poisson regression model. The adjusted hazard ratio (HR) of new-onset stroke between the study and control cohorts was computed using stratified Cox proportional hazard regression (stratified by the age groups of <50 and ≥50 years). Possible confounding factors, such as hypertension, DM, ischemic heart disease (IHD), dyslipidemia, CKD, geographic area, and monthly income, were taken into account prior to the regression study. Kaplan–Meier analysis was also used to calculate the cumulative incidence rate of new-onset stroke in the two cohorts, and the log-rank test was used to analyze differences between the survival curves. A two-sided *p* value of <0.05 was considered significant.

## 3. Results

### 3.1. Patients’ Characteristics

Among gout patients with no history of stroke between 2000 and 2005, we individually compared 15,143 users with and without benzbromarone (Figure 1). The gender and age distributions in the two cohorts are the same (Table 1). There were 0.7% fewer men in the non-benzbromarone cohort than benzbromarone users (69.1% vs. 69.8%). The average age of benzbromarone users is less than 1 year older than non-benzbromarone users (51.7 (SD = 15.79) years compared to 51.37 (SD = 15.90) years). The prevalence of hypertension and ischemic heart disease among users of benzbromarone is higher than that of non-users of benzbromarone.

### 3.2. Risk of New-Onset Stroke between Benzbromarone Users and Benzbromarone Non-Users

**Figure 2:**
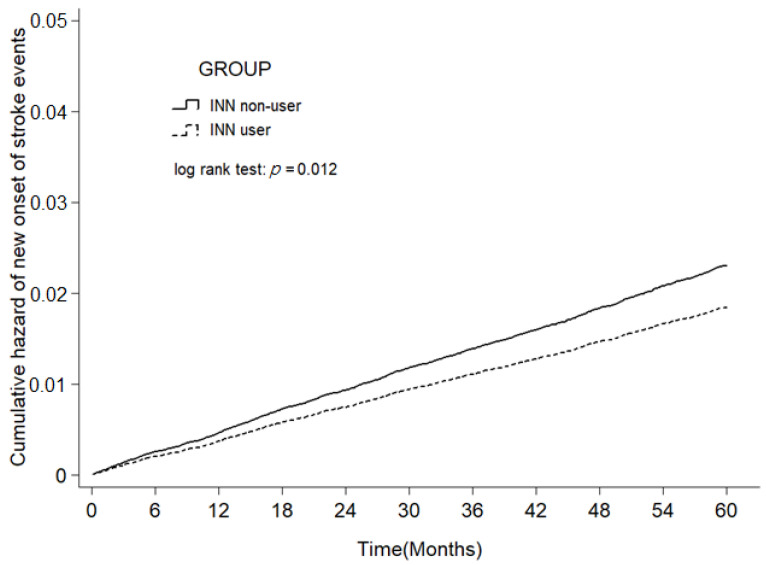
Cumulative incidence of strokes in benzbromarone users (dashed line) and benzbromarone non-users (solid line).

The log-rank test and the cumulative incidence curve of new-onset strokes (Figure 2) showed that the incidence of new-onset strokes in benzbromarone users was significantly lower than that of non-benzbromarone users (*p* = 0.012). During an average follow-up period of 4.75 years, the number of developed new-onset strokes among non-benzbromarone users was 74 more than that of benzbromarone users (645 vs. 571) (Table 2). The average duration before the onset stroke of benzbromarone users (2.55 ± 1.47 years) was significantly longer than that of non-benzbromarone users (2.25 ± 1.44 years; *p* < 0.001; Table 3). The incidence density of new-onset DM among non-benzbromarone users and benzbromarone users was 2.25 and 2.55/10,000 PY, respectively. After adjusting for gender, age, comorbidities, and medications, the risk of new-onset strokes in the benzbromarone user group was reduced by 16% (95% confidence interval [CI] = 0.75–0.94) (Table 2).

### 3.3. Dose–Response Relationship between Benzbromarone Use and the Risk of New-Onset DM

In the benzbromarone users and the control group (non-benzbromarone users), the relationship between the dose of benzbromarone and the risk of new-onset stroke can be shown (Table 2). Among users of benzbromarone, for patients with cumulative DDD > 113, the adjusted HR for new-onset strokes was 0.59 (95% CI = 0.50–0.71). The risk of new-onset stroke risk is significantly reduced due to the higher accumulated DDD of benzbromarone (*p* for trend < 0.001).

### 3.4. Multivariate Analysis

Gender stratification showed that the risk of new-onset strokes among male benzbromarone users was significantly lower than that of non-benzbromarone users (adjusted HR = 0.71, 95% CI = 0.62–0.83; Table 4). The age stratification showed that the risk of new-onset strokes in the subgroup of benzbromarone users aged 40–59 and over 60 was significantly lower than in the corresponding subgroup of non-benzbromarone users (adjusted HR = 0.76 and 0.81 and 95% CI = 0.60–0.97 and 0.71–0.93, respectively). Stratification by comorbidities and multivariate analysis (Table 4) showed that benzbromarone users with hypertension and ischemic heart disease had significantly lower risks of new-onset stroke than benzbromarone non-users with these conditions (adjusted HR = 0.82 and 0.80 and 95% CI = 0.70–0.96 and 0.65–0.99, respectively); similarly, benzbromarone users without hypertension, DM, dyslipidemia, CKD, or IHD had a significantly lower risk of new-onset stroke than benzbromarone non-users without these conditions (adjusted HR = 0.77, 0.75, 0.80, 0.79, and 0.79 and 95% CI = 0.65–0.91, 0.65–0.86, 0.70–0.90, 0.70–0.89, and 0.69–0.91, respectively). After stratification for all factors, the risk of new-onset stroke was lower in benzbromarone users than in benzbromarone non-users.

## 4. Discussion

In our study, the incidence rate of new-onset stroke was lower in patients with gout using benzbromarone than in those without using benzbromarone. The higher the cumulative dose of benzbromarone, the greater the reduction in the risk of new-onset stroke (Table 2). A significantly longer duration before the onset of stroke was observed among benzbromarone users than among benzbromarone non-users (Table 3). After stratification by sex, male benzbromarone users had a significantly lower risk of new-onset stroke than benzbromarone non-users (Table 4). With age stratification, benzbromarone users strikingly reduced the risks of new-onset stroke in the 40–59-year-old and ≥60-year-old subgroups, compared to non-users. The present study revealed that the male patients under benzbromarone (69.8%) were two more times than female ones (30.2%) (Table 1). One animal study revealed that estrogen in the mouse model may regulate the expression or activity of UA transporters, specifically ABCG2 and SLC2A9, and this regulation increases UA excretion via renal proximal tubules and even small intestines [12]. The HepaMeta Study demonstrated that SUA level is related to gender, and higher SUA was noted in Roma males than in females, accompanied by elevated CRP and serum ferritin, as a marker of cardiovascular risk [13]. They pinpointed that females benefited from estrogen itself to prevent hyperuricemia and even gouty attack. This study did not discern a notable difference between female benzbromarone users and non-users (Table 4).

Hyperuricemia is involved in the development and pathogenesis of stroke, atherosclerosis, hypertension, and MetS [14]. Hyperuricemia is related to active xanthine oxidase accompanied by an increase in ROS and a decrease in nitric oxide synthase (NOS). Therefore, hyperuricemia is considered a risk factor for CVE [15]. The imbalance of ROS and NOS leads to endothelial dysfunction [16] and induces plasma renin activity [17]. The Iki Epidemiological Study of Atherosclerosis and Chronic Kidney Disease (ISSA-CKD) study demonstrated that elevated SUA levels are an independent risk factor for new-onset hypertension, especially in the CKD population [18]. Otherwise, in the hypertension population, elevated SUA levels were significantly associated with the prevalence and severity of SCA, especially in the thoracic aorta affected [19]. One Korean NHI study revealed that compared with benzbromarone, allopurinol is associated with an increased risk of combined cardiovascular events and all-cause mortality. Benzbromarone can reduce cardiovascular risk and mortality in patients with gout [20].

The clinical effects of ULAs on CVE are controversial. Some studies demonstrated that allopurinol reduced ROS, delayed the time to onset of angina, and reduced cardiovascular (including stroke)-related mortality [21,22,23]. In contrast, other studies did not support the benefits of allopurinol therapy in gout patients, including beneficial future cardiovascular outcomes [24], and the initiation of xanthine oxidase inhibitors was not associated with a change in the cardiovascular risk [25]. A recent meta-analysis did not find any differences in CVE risks in patients with gout taking ULAs, but the data of uricosuric medications was lacking [8]. In the present study, the risk of new-onset stroke was reduced by 16% with the use of benzbromarone (Table 2). Our study is in accordance with the findings that hyperuricemia plays a key role in IR, MetS, and DM [26]. In addition to hypertension, a recent study suggested that hyperuricemia was related to DM [27].

Insulin may enhance renal urate reabsorption through the stimulation of the urate-anion exchanger URAT1 in the brush border membranes of the renal proximal tubules [28], which can lead to hyperuricemia [16]. Therefore, increasing IR is also a risk factor for hyperlipidemia, which can lead to stroke, cardiovascular accidents, and atherosclerosis [29]. Our previous study with the usage of Taiwanese NHIRD demonstrated that benzbromarone, a URAT1 inhibitor, is related to a reduction in the risk of DM [9]. It is well known that oxidative stress in adipocytes is highly involved in IR and MetS [30], and URAT1 is expressed not only in renal proximal tubular cells but also in adipocytes [31]; therefore, benzbromarone may not only affect uric acid excretion but also improve IR. Another study in an animal model revealed that benzbromarone inhibits both URAT1 and FABP4, which indicates that benzbromarone may be a potential candidate for the treatment of DM and atherosclerosis [32].

Our study demonstrated a reduction in the risk of new-onset stroke with the use of benzbromarone. In subgroup analysis, the risks of new-onset stroke were reduced in male benzbromarone users; the 40–59-year-old; and the ≥60-year-old benzbromarone users. (Table 4). Existing studies have shown that female hormones are associated with lower levels of serum UA via renal clearance [33], and serum UA levels have been found to be significantly associated with prehypertension [34], the carotid-to-femoral pulse wave velocity [35] and cognitive impairment [36] only in men and not in women. In our previous study, male benzbromarone users had a significantly lower risk of new-onset DM than non-users [9]. Above all, male sex and advanced age are both risk factors for gout and its complications. However, due to small subgroup sample sizes, additional large-sample size studies may be needed.

The present study has some limitations. First, it was not a prospective study. Secondly, this study only demonstrated the preventive effects of benzbromarone against stroke in a population with gout and did not investigate its effects in a population without gout. Past studies have shown that UA levels might be linked to the incidences of stroke and MI, but there is no evidence of the potential effects of therapeutic interventions. Thirdly, the NHIRD does not include laboratory data, such as UA levels; therefore, our case definition was based on physician-recorded diagnoses instead of urate crystal identification or the American College of Rheumatology criteria [37]. In our study, gout, and stroke were both accurately diagnosed and coded (ICD-9-CM codes) by specialists according to the standard diagnostic criteria, including the typical symptoms and signs, imaging findings, and laboratory data.

## 5. Conclusions

In this cohort study, the incidence of stroke was lower in benzbromarone users than in non-users in patients with gout. A higher incidence of stroke was observed in the population with gout than in the general population. The prevention of stroke in the population with gout is necessary. Additional epidemiological studies and mechanistic studies are needed to clarify the associations among benzbromarone usage, gout, stroke, atherosclerotic plaque development, and IR.

## Figures and Tables

**Table 1:** Demographic data of INN users and non-users in the gout population (*n* = 30,286).

	INN Non-Users (*n* = 15,143)		INN Users (*n* = 15,143)		
	*n*	(%)	*n*	(%)	*p*
AGE					
<40	3940	(26.0)	3802	(25.1)	0.179
40–59	6370	(42.1)	6421	(42.4)	
≥60	4833	(31.9)	4920	(32.5)	
MEAN ± SD	51.37	(±15.9)	51.77	(±15.8)	
GENDER					
Female	4535	(30.9)	4577	(30.2)	0.599
Male	10,608	(70.1)	10,566	(69.8)	
INSURANCE RANGE					
<NT 15,840	3917	(25.9)	3,99-	(26.4)	0.628
NT 15,840–25,000	6812	(45.0)	6758	(44.6)	
≥NT 25,001	4414	(29.1)	4395	(29.0)	
Mean ± SD	21,641	(±17,838)	21,264	(±17,124)	0.060
COMORBIDITIES					
Hypertension	3820	(25.2)	3978	(26.3)	0.038
DM	1680	(11.1)	1743	(11.5)	0.253
Dyslipidemia	1550	(10.2)	1606	(10.6)	0.292
CKD	152	(1.0)	135	(0.9)	0.313
IHD	1221	(8.1)	1552	(10.3)	<0.001
CUMULATIVE DDD	0	0	134.40	(±193.10)	

Abbreviations. INN, benzbromarone; SD, standard deviation; NT, New Taiwan dollar; DM, diabetes mellitus; CKD, chronic kidney disease; IHD, ischemic heart disease; DDD, defined daily dose.

**Table 2:** Risk of new-onset stroke between benzbromarone users and benzbromarone non-users (*n* = 30,286).

	Case no.	(%)	aHR(95% CI)	*p*	aHR(95% CI)	*p*
Overall	1216	(4.0)				
INN non-user	645	(4.3)	Ref.			
INN user	571	(3.8)	0.84 (0.75–0.94)	0.003		
Cumulative DDDs						
<30	210	(4.2)	1.13 (0.96–1.32)	0.134	Ref.	
30–113	197	(3.9)	0.91 (0.78–1.07)	0.257	0.81 (0.66–0.98)	0.032
>113	164	(3.3)	0.59 (0.50–0.71)	<0.001	0.52 (0.42–0.64)	<0.001
*p* for trend				<0.001		

Values expressed as adjusted hazard ratio (aHR) and 95% confidence interval (CI). Adjusted for age, sex, and comorbidities. Abbreviations are the same as in Table 1.

**Table 3:** Average follow-up duration and average duration of new-onset events of stroke.

	Average Follow-up Duration			Stroke New Onset Average Duration		
	Mean	(SD)	*p*	Mean	(SD)	*p*
Overall	4.75	(0.90)		2.39	(1.46)	
INN non-user	4.69	(1.00)	<0.001	2.25	(1.44)	<0.001
INN user	4.80	(0.78)		2.55	(1.47)	

Unit: year. Adjusted for age, sex, and comorbidities. Abbreviations are the same as in Table 1.

**Table 4:** The risk of new onset of stroke between INN users and non-users adjusted for age, sex and all comorbidities (*n* = 30,286).

	INN Non-User		INN User		INN User vs. Non-INN User	
	No. Cases	(%)	No. Cases	(%)	aHR (95%CI)	*p*
Gender						
Female	199	(4.4)	226	(4.9)	0.99 (0.81–1.19)	0.874
Male	446	(4.20)	345	(3.27)	0.71 (0.62–0.83)	<0.001
Age						
<40	15	(0.38)	9	(0.24)	0.64 (0.27–1.50)	0.303
40–59	153	(2.40)	129	(2.01)	0.76 (0.60–0.97)	0.028
≥60	477	(9.87)	433	(8.80)	0.81 (0.71–0.93)	0.002
Comorbidities hypertension						
No	315	(2.78)	259	(2.32)	0.77 (0.65–0.91)	0.002
Yes	330	(8.64)	312	(7.84)	0.82 (0.70–0.96)	0.016
DM						
No	483	(3.59)	408	(3.04)	0.75 (0.65–0.86)	<0.001
Yes	162	(9.64)	163	(9.35)	0.93 (0.74–1.16)	0.496
Dyslipidemia						
No	562	(4.13)	494	(3.65)	0.80 (0.70–0.90)	<0.001
Yes	83	(5.35)	77	(4.79)	0.82 (0.59–1.13)	0.217
CKD						
No	638	(4.26)	559	(3.72)	0.79 (0.70–0.89)	<0.001
Yes	7	(4.61)	12	(8.89)	1.95 (0.70–5.41)	0.200
IHD						
No	466	(3.35)	385	(2.83)	0.79 (0.69–0.91)	0.001
Yes	179	(14.66)	186	(11.98)	0.80 (0.65–0.99)	0.038

Values expressed as adjusted hazard ratio (aHR) and 95% confidence interval (CI). Abbreviations are the same as in Table 1.

## Data Availability

Data may be available upon request to interested researchers. Please send data requests to: Shang-Jyh Hwang, Professor. Division of Nephrology, Department of Internal Medicine, Kaohsiung Medical University Hospital, Kaohsiung Medical University.
